# A survey of contextual factors and psychological needs satisfaction as correlates of youth athletes’ developmental outcomes in the Ethiopian sports academy context

**DOI:** 10.1186/s13102-022-00545-8

**Published:** 2022-08-16

**Authors:** Tefera Tadesse, Aemero Asmamaw, Sirak H/Mariam, Beshir Edo

**Affiliations:** 1 Institute of Educational Research, Addis Ababa University, PO Box 1176, Addis Ababa, Ethiopia; 2 Institute of Medical Education, University Hospital, LMU Munich, 80336 Munich, Germany; 3 Department of Psychology, The University of Gondar, Gondar, Ethiopia; 4 Kotebe University of Education, Addis Ababa, Ethiopia; 5 Addis Ababa Science and Technology University, Addis Ababa, Ethiopia

**Keywords:** Contextual environment, Ethiopia, Developmental outcome, Psychological needs satisfaction, Sports academy

## Abstract

**Background:**

This study examined the contextual factors associated with psychological need satisfaction (PNS) and the predictability of the PNS components, together with the contextual factors, on the developmental outcomes of elite young athletes in the Ethiopian sports academies, and further identified differences in perception of PNS from a comparative perspective. The study used a cross-sectional survey design applying developmental and PNS theories as guiding frameworks. Samples of elite young Ethiopian athletes participated (n = 257, 47.47% were women with a mean age of 17.44 years and SD = 0.87, and 52.53% were men with a mean age of 18.25 years and SD = 1.14).

**Results:**

Structural equation modeling showed that the three PNS domains, together with the five contextual factors positively predicted the three developmental outcomes measured (41–54% explained variance). Moreover, there were higher differences in PNS (0.55 ≤ Cohen’s d ≥ 0.71) among young athletes classified by the sport types.

**Discussion:**

As per the findings of this study, young athletes may differ in the levels of PNS they obtained depending on the type of sports enrolled in sports academies. Also, the results of this study indicated that PNS attained may be modestly influenced by some contextual factors. It also evidenced that those developmental outcomes in elite young athletes are significantly positively associated with contextual and PNS factors. Stakeholders such as young athlete coaches, parents, sports psychologists, and administrators must consider the differing implications of program type during the elite young athletes’ participation in sports academics and the significant positive association between contextual factors, PNS, and developmental outcomes of elite young athletes.

**Conclusions:**

In sum, the PNS of youth athletes may differ across sports types and the talent development of elite young athletes should emphasize the individual nature of the processes. Also, it can be concluded that the PNS components than the contextual factors had higher predictions of developmental outcomes.

## Background

The notion of *sport for development* refers to the use of sport, or any form of physical activity, to provide children and adults with the opportunity to achieve their full potential through programs that promote not only their subjective-wellbeing [1], but also contributes more positively, towards their personal and social development [2]. In this sense, participation in sports is a potentially important arena for the developmental outcome of youths [3]. In recent years, there has been increasing attention and commitment to organized sports for youth well-being and development. The underlying premise guiding these interest and commitment is that youth’s participation in sports affords multiple psychological and social benefits [4].

The growing approach to youth research and practice, which is called positive youth development (PYD) Damon [5] has been associated with indicators commonly known as the 5Cs including, competence, confidence, connection, character and caring [6]. Research indicated that the sport education environment, characterizing sport-based positive youth development [7], is a salient context for developing the 5Cs [8, 9].

Moreover, sports participation has been linked with a range of educational and developmental benefits such as higher academic performance in high school, greater likelihood of attending college, and greater autonomy and satisfaction with job experience [10]. Also, many psychosocial health benefits in youth are attributed to sports participation [11].

Enduring and healthy participation in any sport is more likely when the participant is optimally functioning and experiencing subjective well-being [12]. *Psychological needs satisfaction* (PNS) or life satisfaction is the cognitive component of subjective well-being and plays an important role in developmental outcome [13]. Study findings show that the existence of support systems, engagement in optimally challenging activities, the happenstance of positive life events, and high-quality interactions with significant others contribute to the development of PNS [14]. Through a well-designed sports education programs, young athletes experiences more positive than negative behaviors [11], and uses own strengths to fulfill pursuits and contribute to society [15]. As a result, he/she leads a healthy, satisfying, and productive life as a youth, and later as an adult [16].

PNS is one of the essential ingredients of young athlete’s participation and commitment in sports education and sports performance. The PNS theory is a theory of motivation, as Deci and Ryan [17] describe the ‘‘energy, direction, persistence… aspects of activation and intention’’ that addresses the why of human behavior. Hence, motivation is vital for learning and development in sports. Numerous studies have confirmed that intrinsic or more internalized forms of motivation are associated with increased interest, engagement, effort, learning, and satisfaction in life [18]. Deci and Ryan [19] identified three basic needs conducive to the development of highly internalized motivation. These are autonomy, competence, and relatedness.

*Autonomy* is an internal perceived locus of causality, which has the ability to posit that choice and autonomy enhance intrinsic motivation [20]. *Competence* is conceptualized as a sense of self-efficacy, and it is an important tool in any motivating scenario because people adopt activities that make them feel their actions affect outcomes. *Relatedness* is described as ‘‘the need to feel belongingness and connectedness with others’’ [21]. The desire for interpersonal attachments is a fundamental human motivation, which is inherently intrinsic in nature [22]. Yet, research findings support that involvement in sports associated with higher developmental outcomes [23], and satisfaction depends upon a variety of programmatic and contextual factors [8], and on school and family support systems [9].

Youth athlete behaviors such as psychological readiness, attendance to the regular program, and time spent for personal training are critical factors for success during the academy years. The term “psychological readiness” describes a set of behaviors and thoughts that youth athletes engage in prior to a scheduled performance of a specific sport skill or competition. Psychological readiness is effective as it helps young athletes to focus on task-relevant information and block out distractions, thus increasing young athletes’ concentration and confidence [6]. Also, the level of attendance to a regular scheduled sports program indicates the level of interest the young athlete has for that program. Research has indicated that the time youth spend in their sports participation, play a role in how sports participation is linked to youth development [24]. Most often, youth coaches provide valuable feedbacks on the quality of young athletes performance only during ongoing practice [25]. Hence, regularly attending a scheduled performance session is very important.

Young athletes with a “hard work ethic” commonly possess a vision of what it takes to succeed and spend extra time for their own sports training [26]. Thus, they consistently invest high levels of effort into training over a prolonged time [16]; sacrifice their social lives; and accept challenges without giving up or dropping out of the sport they want to excel in [27].

### Rationale

The term developmental outcome has captured the general sense of desirable consequences in youth as a result of structured programs [16]. Over the last decade or so, there has been increasing interest in how organized youth sports can foster positive developmental outcome [23]. In view of this, more attention on developmental outcome and its correlates in sports academies seems justified. Sport may be associated with improved psychosocial health above and beyond improvements attributable to participation. Specifically, team sport is associated with improved health outcomes compared to individual sports [15]. Furthermore, athletes playing individual sports had higher scores on "planning" and "effort" than team sport athletes, highlighting the importance of differences between types of sport [28, p. 901].

PNS-based research has shown that satisfying youths’ psychological needs may promote positive developmental outcomes [29]. Participation in organized sport has been linked with a range of educational and developmental benefits [10, 30]. For example, a study found that satisfaction of young swimmers’ needs for competence was positively related to their developmental experiences [31]. However, there is minimal research investigating the benefits of elite young athletes’ participation in organized sports in the sports academies setting.

In the present study, we focused on three developmental outcomes that may be significant for youth athletes better psychosocial functioning and happiness. First, we considered gains in personal and social development, as well as, gains in higher-order cognitive skills because these are key facets of affective outcomes for youth to lead a healthy, satisfying, and productive life as a youth, and later as an adult [15, 16]. Second, as overall satisfaction is integral to successful psychosocial functioning and sports performance [23], we examined the extent to which young athletes are satisfied with the developmental experiences they have had in the sports academies. Moreover, given that feeling of PNS has been highlighted as a predictor of developmental outcome in earlier studies of adolescents and youths [29, 32], we focused on the measurement of PNS as well as the extent of its relationships with developmental outcome. As variations in sport type (team vs. individual sports) [15], as well as gender impact youth sports experiences [33], we also studied PNS and its relations with developmental outcome from these perspectives.

In many areas of sport, young people differ from one another in their accomplishments and in the affluence with which they achieve performance excellence. Empirical evidence supports varying degrees of innate talent among individuals [34]. Also, the individual difference in athletic performance may result from variations in the mechanism and processes leading to performance excellence. As Howe, Davidson [35] argues ‘differences in early experiences, preferences, opportunities, habits, training, and practice are the real determinants of performance excellence in many areas of expertise, ranging from music, dance, art, and literature to sports, and others’ (p. 399). In line with the discussion, young athletes’ sports excellence requires both talent and a system to establish a conducive environment to maximize young athletes’ development to the elite level [36].

Most of the literature addressing talent identification and development in elite young athletes has focused on physical and physiological factors and how these relate to age and maturation [37–41]. This focus confines talent development as an exclusive function of physical and physiological factors, leaving aside the multifaceted and dynamic nature of sports talent and the potential contributions of other factors. Regardless of this, athlete developmental pathways are supported and restricted by the contextual-social environment and psychological characteristics. As Bronfenbrenner and Ceci [42] expressed, 'if proximal processes [such as individual-environment interactions] are the engines of development, it is the characteristics of (the) person and context that provide the needed fuel' (p. 584). This implies that factors related to elite athletes’ talent development should be analysed from multiple perspectives.

There remains a consensus that traditional cross-sectional talent identification models are likely to exclude many, particularly those late maturing, ‘promising’ children from development programs. Guth and Roth [43] propose a conceptual framework that acknowledges the mutual influences of genetic and environmental factors on sports talent. According to this framework, sport talent identification and development should be dynamic and interconnected, considering the contributions of genetics and environment and the potential to develop. However, the relative importance of genetic versus environment factors on athletic success likely varies widely between sports as every sport has unique physical requirements and these requirements maybe dramatically peculiar for one sport than others [44]. Hence, there is a need for optimizing genetically driven physical and mental traits with the ideal environment for the specific sport [45].

In line with this, a researcher clarifies dynamical systems theory as a multidisciplinary theoretical rationale for capturing how multiple interacting constraints can shape the development of sporting talent among elite young athletes [46]. According to this theory, young athletes’ talent development programs should emphasize the individual nature of talent development pathways to sporting excellence and identify the range of interacting factors that impinges on the sporting potential of individual athletes. This could mean that elite athletic performance is the cumulative result of ‘a favourable genetic profile of an athlete, combined with an optimal training environment’ [43, p. 1].

Regardless of this, studies on the talent identification and development pathway of young athletes have tended to be, in the words of Phillips, Davids [46], ‘mono-disciplinary, typically adopting genocentric or environmentalist positions’ (p. 271). Accordingly, talent development programs emphasize the notion of common optimal performance models, capitalizing on the importance of current performance on anthropometric and physical characteristics tests referenced to group norms [47]. This is against the contemporary perspective that emphasizes the complex nature of sports talent [48] and the range of interacting factors influencing the sporting potential of individual athletes [37].

Moreover, little attention has been paid in the literature to the other factors such as contextual and psychological factors, the limited studies identified that these factors together significantly influence the talent development of elite young athletes [49, 50]. For example, considering the multidimensionality of sports talent, Tribolet, Bennett [51] tested a multidimensional talent selection approach. The results show significant age-related differences in Australian Football players' anthropometry, fitness, and coach skill ratings. Also, Murr, Feichtinger [52] conducted a systematic review and found results supporting the predictive validity of some psychological variables for young soccer players. As the authors reported, those results complement Murr, Raabe [53] previous review of the predictive value of physical and physiological characteristics.

In line with the discussion, in this study, we focused on the capacity of elite young athletes and the psychological and contextual factors that underpin this process [54]. Therefore, the purpose of this study was to examine the contextual factors associated with the PNS and the predictability of the PNS components, together with the contextual factors, on the developmental outcomes of elite young athletes in the Ethiopian sports academies, and further discover differences in the perception of PNS from a comparative perspective. More specifically, the study addresses the following three research questions:

1. Do young athletes differ in the levels of PNS attained depending on the kind of sport types enrolled in the Ethiopian sports academy context?
2. Do contextual factors predict PNS among young athletes in the Ethiopian sports academies?
3. Do higher levels of PNS predict higher levels of developmental outcomes after accounting for the difference in the contextual factors among young athletes in Ethiopian sport academy?

## Methods

### Study design

This study used a cross-sectional survey design collecting quantitative data from a sample of 257 Ethiopian young athletes. By assessing developmental outcomes, contextual factors, and PNS components involved in the process, this study tried to portray a more complex picture of [55].

### Conceptual model

**Fig. 1:**
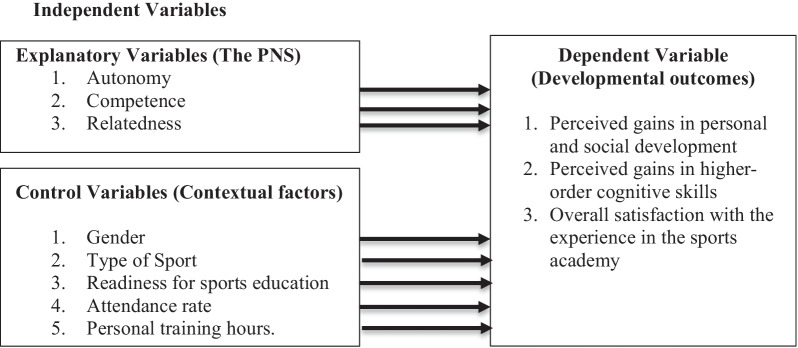
The conceptual model illustrating the relationships between the independent variables and dependent variables measured in the study. *Note:* PNS = Psychological Need Satisfaction

The conceptual model of this study consisted of three PNS components, along with five contextual factors, and three developmental outcomes. Figure 1 presents the variables included and the directional relationships found between them.

As shown in Fig. 1, the PNS components and the contextual factors serve as independent variables and the developmental outcomes as dependent variables. The PNS variables defined in the model are postulated as comprising of the three components of PNS, including autonomy, competence, and relatedness, as suggested in the literature on this field [17, 19, 56]. The control variables consist of five contextual factors including gender, sport type, readiness, attendance, and personal training hours. With the help of this model, in this study, the authors emphasized the developmental and psychological needs of young elite athletes, and tested the relationships of contextual factors and the PNS components with developmental outcomes among young athletes in sports academy setting.

### Population and sampling

The study population included all elite young athletes enrolled in the Ethiopian sports academics during the academic year. The Ethiopian sports academies are in their early stages of development as centers of education (learning) and sports [57]. The present study randomly sampled from two of five sports academies in existence in Ethiopia. One sport academy is situated in the capital Addis Ababa and the other in Asella town, Arsi Zone, Oromiya Regional State, Ethiopia. The final sample included young athletes (*n* = 257) enrolled in these two institutions, and we categorized the enrolment data by gender and age, in order to determine participation rate. Accordingly, from the total of 257 sample participants, 47.47% were women and 52.53% were men. The mean age of the women sample was 17.44 years with SD = 0.87 and that of men sample was 18.25 years with SD = 1.14. From these figures it is clear that the men sample participants were relatively older than the women sample participants.

Data from this sample has been published elsewhere [58]. However, the published article and this paper completely differ in substantive contents. While the published article emphasized on reporting the reliability and validity of the instrument to reveal its psychometric properties and another article, this paper focuses on the relationships of youth athletes’ demographic and contextual factors, psychological needs satisfaction, and development and satisfaction.

### Data collection tools

The relevant data for this study was collected through a self-reported questionnaire. The questionnaire has been adapted to the existing sports academy context through validation processes, including experts’ reviews, pilot testing, and reliability tests [58]. As part of the validation process, the study instrument was modified to fit with the existing sports academy context, by translating the study questionnaire, which was originally prepared in English, to a local national language called, “Amharic”. A language expert who was fluent in the source language and native in the target language conducted the translation.

The demographic factors include those characteristics such as age and gender, as well background information such as prior educational achievements, and current experiences (the type of sport enrolled and grade year). The contextual data were secured through the young athlete self-report. In addition, young athlete readiness for sports education during the academy season was measured with a single item, along with other items that ask the rate of attendance in the regular academy season and personal training experiences.

In the present study, the researchers used the 14 items from the Basic Need Satisfaction Scale adapted to reflect the sports academy context. The items were answered using a 4-point scale anchored by 1 (never) and 4 (always). Example items are “The tasks I have to do at my sport type are in line with what I really want” (autonomy), “I feel competent at my sporting activities” (competence) and “I feel like I am part of the group” (relatedness). In an international published article, researchers reported acceptable factorial validity and internal consistency with the same sample of sport academy young athletes [58]. In line with that, we used a three factor PNS factors to produce three composite needs satisfaction scores representing autonomy, competence, and relatedness. Hence, PNS was measured in a previous study of the same sample, but that was for examining the psychometric properties instead of measuring the relationships of PNS with developmental outcomes.

The three development outcomes (i.e., gains in personal and social development, gains in higher-order cognitive skills, and overall satisfaction with the experience in sports academy) were measured using subscales from the modified learning gains and satisfaction survey [59]. The perceived gains and satisfaction survey were completed by the participants at the end of the regular academy season to evaluate the young athletes’ positive developmental outcomes through their involvement in sports education at their corresponding sports academy. The survey has 14 items and evaluates three dimensions: (a) gains in personal and social development. Youth sport academy participants were asked to reflect on their current sport education involvement in their respective sports academy and respond to each statement using a 4-point Likert-type scale.

Each subscale contained 4–5 items representing experiences that occurred during their sport education involvement. The gains scale items were answered using a 4-point scale anchored by 1 (very little) and 4 (very much). Example items are “Working effectively with others” (gains in personal and social development) and “Solving complex real-world problems” (gains in higher-order cognitive skills). Similarly, the satisfaction items were answered using a 4-point scale anchored by 1 (never true) and 4 (very true). Example item is “My colleagues are happy with the sports education offered in our sports academy”. These scales obtained acceptable internal consistency in the same sample of young athletes (0.80 ≤ *α* ≥ 0.88).

### The study procedures

Prior to the data collection, the researchers obtained Ethics Committee approval from Ethiopian Youth Sports Academy. Data collection was held after obtaining permission from the required senior managers, and after getting informed consent from the assigned caregivers and each individual young athlete. Data collection was held after obtaining permission from the required senior managers, and after getting oral consent from the assigned caregivers. Before asking informed consent, the data collectors briefly presented the study information, regarding the objectives of the study, the volunteer nature of participation, and the expected results to aid in their decisions to participate. Once informed consent secured, participants completed study instrumentation to test objectives.

### Data analysis methods

The data for the present study were analyzed in two ways, including group difference tests and multiple regression analysis models using SEM. All analyses were run using stata 15 data analysis and statistical software package. The main intention of our analyses was to draw conclusions at the level of the individual, not the institution level, so our report chiefly focuses on individual-level effects.

Across all analyses, sport type was dummy coded with athletics and indoor sports and cycling entered the models and team sports as the reference group. In addition, gender was dummy coded with the male entered the models, and female as the reference group. In addition, the researchers used partial correlation analyses, as a preliminary step, to identify potential contextual factors of PNS and developmental outcome in sports academies.

In this study, we run group comparison tests, as a first step, on the PNS measures to examine the pattern of significant differences between sample groups classified by their sport types enrolled in the sports academies studied (Research question 1). For this, we used one-way ANOVA for the total sample. Then, two sets of analyses were performed. The first set used multiple regressions to examine, the relative influence of contextual variables on young athlete PNS variables (Research question 2). The second set used two-step multiple regressions to assess the relative prediction of the PNS variables on developmental outcome, after accounting for the differences in the prediction of the contextual variables (Research question 3).

## Results

The findings of this study are presented in two parts. Part 1 provides the group comparison results included in the study. Part 2 presents the results of multiple regression analyses.

### Results of group difference tests

A one-way between subjects’ ANOVA was conducted to compare the effect of sports type on perceived autonomy for the young athlete groups in team sports, athletics, and indoor sports and cycling conditions. Table 1 presents the means and standard deviations for young athletes across sport type.

**Table 1:** Perceived PNS between youth athletes enrolled in Team sports, Athletics, and Indoor sports and cycling

Variable	Team sports (n = 93)		Athletics (n = 137)		Indoor sports and cycling (n = 27)		*95% CI*		*df*	*F value*	*Cohen’s d*
	*M*	*SD*	*M*	*SD*	*M*	*SD*	LL	UL			
Autonomy	2.98	0.81	2.85	0.82	2.45	0.93	0.08	0.97	(2, 254)	4.25*	0.64
Competence	2.99	0.77	2.90	0.80	2.55	0.94	0.01	0.87	(2, 254)	3.16*	0.55
Relatedness^a^	2.83	0.74	2.74	0.85	2.24	0.93	0.11	1.01	(2, 254)	5.38**	0.71
Relatedness^b^	2.83	0.74	2.74	0.85	2.24	0.93	0.03	0.97	(2, 254)	5.38**	0.60

Effect size δ is defined as the ratio of the difference between the mean scores of young athletes enrolled in sporting events over the pooled standard deviation, δ = (μ1—μ0)/σ

M, mean; SD, standard deviation; CI, 95% confidence interval; LL, lower limit; UL, upper limit; df, degrees of freedom; F value, a value on the F distribution; Cohen’s d, effect size

*p < .05, **p < .01

^a^Between youth athlete in team sports versus indoor sports and cycling

^b^Between youth athlete in Athletics versus indoor sports and cycling

There was a significant effect of sport type on perceived autonomy at the *p* < 0.05 level for the three events conditions [*F*(2, 254) = 4.12, *p* = 0.015]. Post hoc comparisons using scheffe test indicated that the mean score for the team sport youth group (M = 2.98, SD = 0.81) was significantly different from the youth group mean in the indoor sports and cycling condition (M = 2.45, SD = 0.93). However, the athletics youth group (M = 2.85, SD = 0.82) did not significantly differ from the youth groups in team sports and indoor sports and cycling conditions.

In a similar vein, a one-way between subjects’ ANOVA was conducted to compare the effect of sport type on perceived competency of the same sample. There was a significant effect of sport type on perceived competency at the *p* < 0.05 level for the three sport types [*F*(2, 254) = 3.16, *p* = 0.044]. Post hoc comparisons using scheffe test indicated that the mean score for the young athlete in team sport condition (M = 2.99, SD = 0.77) was significantly different from the young athlete mean in the indoor sports and cycling condition (M = 2.55, SD = 0.94). However, the young athletes in athletic sports (M = 2.90, SD = 0.80) did not significantly differ from the team sports and indoor sports and cycling conditions.

To examine whether the same applies for the difference in perceived relatedness, the same one-way between subjects ANOVA was conducted across the three group conditions. The result was pretty much the same. There was a significant effect of sport type on perceived relatedness at the *p* < 0.05 level for the three sport types [*F*(2, 254) = 5.38, *p* = 0.005]. Post hoc comparisons using Games-Howell test (homogeneity of variance violated) indicated that the mean score for the young athletes in team sports group (M = 2.83, SD = 0.74) was significantly different from the group mean in indoor sports and cycling condition (M = 2.24, SD = 0.93). Similarly, the mean score of young athletes in athletics (M = 2.74, SD = 0.85) was significantly different from the young athlete group mean in indoor sports and cycling condition (M = 2.45, SD = 0.93). However, the athletics youth group did not significantly differ from the team sports conditions.

Looking at the Cohen’s d scores of the F-value test for each PNS variable in Table 1, it is clear that young athletes enrolled in indoor sports and cycling had less perceived PNS compared to young athlete enrolled in team sports and athletics, and the mean differences based on Cohen’s d scores was ≥ 0.55, which are medium and large effect sizes [60].

Taken together, these results suggest that differences in sport type really do influence PNS of young athletes. Specifically, our results suggest that when young athletes enrolled in indoor sports and cycling, they perceived less PNS. However, it should be noted that young athletes enrolled in team sports do not appear to be significantly, different in the perceived PNS level with youth groups enrolled in athletics.

### Results of multiple regression analyses

Multiple regression analysis was conducted to examine the relationship between the three PNS components and the five contextual factors as predictors (independent variables). Table 2 presents the summary results of the regression models predicting the three components of PNS by the five contextual factors.

**Table 2:** Summary of regression models predicting the three components of PNS

Dependent variable	Independent variable	B	SE^a^	t value	*p*	*β*	*F* value	R^2^
Autonomy	Gender	− 0.11	0.09	− 1.23	0.2180	− 0.07	5.78***	.08
	Sport type	− 0.22	0.07	− 3.03	0.0030	− 0.18**		
	Attendance rate	0.10	0.04	2.68	0.0080	0.16**		
	Personal training hours	0.11	0.04	2.51	0.0130	0.15*		
Competence	Gender	− 0.18	0.08	− 2.18	0.0300	− 0.13*	7.05***	.10
	Sport type	− 0.20	0.07	− 2.97	0.0030	− 0.18**		
	Attendance rate	0.09	0.03	2.67	0.0080	0.16**		
	Personal training hours	0.12	0.04	2.90	0.0040	0.17**		
Relatedness	Gender	− 0.20	0.08	− 2.46	0.0150	− 0.15*	7.14***	.10
	Sport type	− 0.20	0.06	− 3.15	0.0020	− 0.19**		
	Attendance rate	0.09	0.03	2.71	0.0070	0.16**		
	Personal training hours	0.10	0.04	2.51	0.0130	0.15*		

^a^Standard Error, B, unstandardized beta coefficient; *β*, standardized beta coefficient; t value, the size of the difference relative to the variation; F value, a value on the F distribution; R^2^, the goodness of fit of a model

*p < .05. **p < .01. ***p < .001

As can be seen from Table 2, the last column, the variabilities accounted by the three models ranges between 0.08 and 0.10, which means that 8–10% of the total variance in young athlete perceived PNS has been explained by the model containing gender, sport type, attendance, and personal training hours. (Model1 R^2^ = 0.08, *F*[4, 252] = 5.78, *p* < 0.001; Model2 R^2^ = 0.10, *F*[4, 252] = 7.05, *p* < 0.001, and Model3 *R*^2^ = 0.10; *F*[4, 252] = 7.14, *p* < 0.001). This is not very impressive, but not bad either compared with the R^2^ values one tends to get in analyses of survey data examining contextual factors. In addition, it is clear from Table 3 that both sport type and attendance rate consistently predicted relatively higher across the three PNS measured (0.16 ≤ *β* ≥ 0.19).

**Table 3:** Summary of the 2-step hierarchical regression model predicting gains in personal and social development (n = 257)

Dependent variable	Independent variable	B	SE^a^	t value	*p*	*β*	F value	Adj R^2^
*Step one*								
Gains in personal & social development	Autonomy	− 0.07	0.12	− 0.57	0.567	− 0.08	89.00***	.51
	Competence	0.49	0.20	2.41	0.017	0.47*		
	Relatedness	0.36	0.13	2.71	0.007	0.33***		
*Step two*								
Gains in personal & social development	Gender	− 0.12	0.06	− 1.92	0.056	− 0.08	36.16***	.54
	Sport type	− 0.09	0.05	− 1.8	0.074	− 0.08		
	Readiness	0.05	0.06	0.92	0.357	0.04		
	Attendance rate	0.06	0.03	2.30	0.022	0.10*		
	Personal training hours	0.01	0.03	0.37	0.713	0.02		
	Autonomy	− 0.07	0.12	− 0.56	0.576	− 0.07		
	Competence	0.47	0.20	2.34	0.020	0.45*		
	Relatedness	0.32	0.13	2.44	0.015	0.30*		
							Δ Adj R^2^	.03

^a^Standard Error, readiness, readiness for academic sports education; B, unstandardized beta coefficient; *β*, standardized beta coefficient; t value, the size of the difference relative to the variation; F value, a value on the F distribution**;** R^2^, the goodness of fit of a model

*p < .05. **p < .01. ***p < .001

**Fig. 2:**
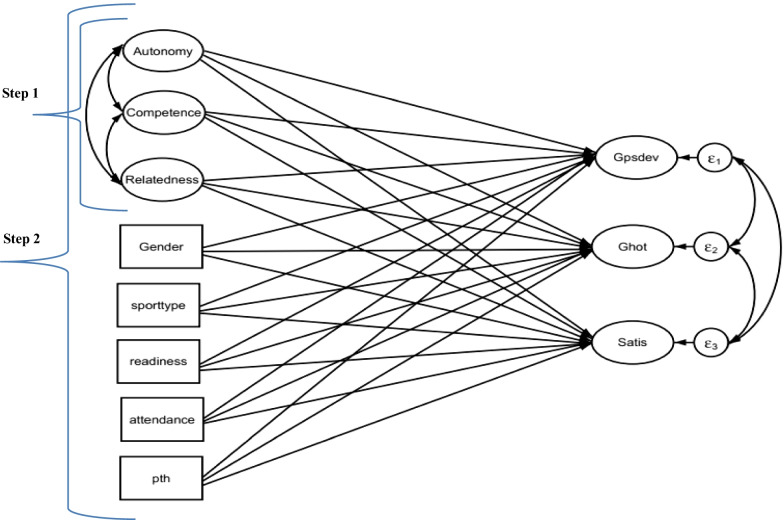
The two-step hierarchical regression models of the PNS variables and contextual factors predicting the developmental outcomes. *Note:* pth = personal training hours

Similar multiple regression analyses were conducted to examine the relationship between developmental outcome and those potential predictors, comprising of the PNS components and the contextual factors. In the two-step hierarchical regression model, the first step consisted of the three PNS components as predictors of the three developmental outcomes as indicated in Fig. 2, step 1. The second step comprised of the PNS components, together with, the five contextual variables as predictors of developmental outcomes as shown in Fig. 2, step 2.

As can be seen in Fig. 2, step one, the regression models are drawn from each PNS component to each developmental outcome variable. The second of the two-steps, regression models include the three PNS variables, together with five contextual factors as predictors of developmental outcome. Table 3 presents the summary of the 2-step hierarchical regression models predicting personal and social development gains.

As shown in Table 3, in the first step, the contextual factors statistically predicted young athletes’ gains in personal and social development, when entered into the regression models (Step 1: Model *Adj. R*^*2*^ = 0.51, *F*[3, 253] = 89.00, *p* < 0.001). Similarly, when the PNS variables were added to the regression models, they brought significant changes in predictions of the outcome variable of interest. Step 2: Model *Adj. R*^*2*^ = 0.54, ∆R^2^ 0.40, *F* Change [8, 248] = 36.16, *p* < 0.001. It is clear from these results that the inclusion of the PNS variables resulted in substantial changes in capacity to predict the measured outcome (∆R^2^ = 0.03). In the first step, the PNS components had strong relationships on perceived gains in personal and social development outcomes (0.33 ≤ *β* ≥ 0.47, *p* < 0.001). In the second step, only the attendance rate has a significant positive relationship on perceived gains in personal and social development outcome, but the other contextual factors were non-significance when added to the equation (Table 3). In these regression models, two of the three PNS variables, had a higher positive effect (0.30 ≤ *β* ≥ 0.45, *p* < 0.05) on the gains in personal and social development outcome.

To determine whether the same mechanism holds for perceived gains in higher-order cognitive skills, these two-steps analyses were repeated using the same samples. Table 4 presents the summary of the 2-step hierarchical regression models predicting gains in higher-order cognitive skills.

**Table 4:** Summary of the 2-step hierarchical regression model predicting gains in higher-order cognitive skills (n = 257)

Dependent variable	Independent variable	B	SE^a^	t value	*p*	*β*	F value	Adj.R^2^
*Step one*								
Gains in higher-order cognitive skills	Autonomy	− 0.17	0.14	− 1.27	0.207	− 0.18	67.21***	.44
	Competence	0.43	0.22	1.97	0.050	0.41^ms^		
	Relatedness	0.48	0.14	1.22	0.001	0.43***		
*Step two*								
Gains in higher-order cognitive skills	Gender	0.00	0.07	0.00	0.997	0.00	26.62***	.46
	Sport type	− 0.08	0.06	− 1.41	0.160	− 0.07		
	Readiness	0.05	0.06	0.90	0.371	0.04		
	Attendance rate	0.05	0.03	1.83	0.068	0.09		
	Personal training hours	0.05	0.03	1.42	0.156	0.07		
	Autonomy	− 0.19	0.14	− 1.40	0.163	− 0.20		
	Competence	0.43	0.22	1.93	0.055	0.40^ms^		
	Relatedness	0.46	0.14	3.16	0.002	0.41**		
							Δ Adj R^2^	.02

^a^Standard Error, readiness, readiness for academic sports education; B, unstandardized beta coefficient; *β*, standardized beta coefficient; t value, the size of the difference relative to the variation; F value, a value on the F distribution; R^2^, the goodness of fit of a model

*p* < .10 = marginally significant (ms), *p < .05. **p < .01. ***p < .001

As shown in Table 4, in the first step, the PNS components statistically predicted young athletes’ gains in higher-order cognitive skills (Step 1: Model *Adj. R*^*2*^ = 0.44, *F*[3, 253] = 67.21, *p* < 0.001). Similarly, when the contextual factors were added to the regression models, they brought significant changes in predictions of the outcome variable measured. Step 2: Model *Adj. R*^*2*^ = 0.46, ∆R^2^ = 0.02, *F* Change [8, 248] = 26.62, *p* < 0.001. Looking into the *β* coefficients, it is clear that, first the PNS components (relatedness and competency) had strong relationships (0.40 ≤ *β* ≥ 0.43, *p* < 0.05). In the second step, for all contextual factors the relationships significantly increased, meaning the contextual factors were related to perceived gains in higher-order cognitive outcomes when added to the regression equations. In this regression model, only one of the PNS variables, relatedness had a higher positive effect (*β* = 0.41) on gains in higher-order cognitive skills outcome (Table 4).

In a similar vein, to determine whether the same mechanism holds for perceived satisfaction outcome, the same two-steps analyses were repeated using the same samples. For these, results were pretty the same. Table 5 presents the summary of the 2-step hierarchical regression models predicting satisfaction.

**Table 5:** Summary of the 2-step hierarchical regression model predicting satisfaction (n = 257)

Dependent variable	Independent variable	B	SE^a^	t value	*p*	*β*	F value	R^2^
*Step one*								
Satisfaction	Autonomy	− 0.10	0.14	− 0.73	0.468	− 0.11	46.86***	.36
	Competence	0.35	0.23	1.53	0.127	0.34		
	Relatedness	0.39	0.15	2.61	0.010	0.37*		
*Step two*								
Satisfaction	Gender	− 0.12	0.07	− 1.74	0.084	− 0.09	21.41***	.41
	Sport type	− 0.12	0.06	− 2.18	0.030	− 0.11*		
	Readiness	0.13	0.06	2.12	0.035	0.11*		
	Attendance rate	0.06	0.03	1.93	0.055	0.10		
	Personal training hours	0.07	0.03	1.92	0.056	0.10		
	Autonomy	− 0.11	0.14	− 0.76	0.446	− 0.11		
	Competence	0.31	0.22	1.39	0.166	0.30		
	Relatedness	0.35	0.15	2.42	0.016	0.33*		
							Δ Adj R^2^	.05

Standard Error, readiness, readiness for academic sports education; B, unstandardized beta coefficient; *β*, standardized beta coefficient; t value, the size of the difference relative to the variation; F value, a value on the F distribution

*p < .05. **p < .01. ***p < .001

As shown in Table 5, in the first step, the contextual factors statistically predicted young athletes’ satisfaction, when entered into the regression models (Step 1: Model Adj. *R*^*2*^ = 0.35; *F*[3, 253] = 46.86, *p* < 0.001). Similarly, when the control variables were added to the regression model, they brought significant changes in the predictions of the response variable satisfaction. Step 2: Model Adj. R^2^ = 0.41, ∆R^2^ = 0.25, *F* Change [8, 248] = 21.41, *p* < 0.001. It is clear from these results that the inclusion of the PNS variables resulted in substantial changes in capacity to predict the satisfaction outcome.

First, the three PNS variables had positive relationships on satisfaction outcome (0.14 ≤ *β* ≥ 0.21, *p* < 0.05). In the second step, for three of the five contextual factors relationships was found non-significance. In this regression model, only the PNS variables, particularly relatedness had a higher positive effect (*β* = 0.33) on satisfaction outcome (Table 5).

Overall, similar to the anticipated predictions, this PNS-outcome relation was minimally affected by the five contextual factors. The relationship continued to be strong, for the most part, that is, the PNS components first independently predict the developmental outcome, and those relationships continue to exist when the controlling variables were added to the regression equations. The PNS relationships with developmental outcome (0.30 ≤ *β* ≥ 0.45, *p* < 0.05) were significantly higher than those relationships made by the contextual factors (0.12 ≤ *β* ≥ 0.21, *p* < 0.05). Comparing the relationships accounted for each PNS variables, the relatedness variable made greater and more consistent predictions on the developmental outcome measured (0.30 ≤ *β* ≥ 0.41, *p* < 0.05).

In general, these results suggest that most of the contextual factors do not leverage the relationships between PNS and developmental outcome, if they do the relationship was found weak or minimal. It is also clear that the inclusion of the PNS variables substantial sustain capacity to predict the three measured outcomes, with the relatively highest prediction in gains in personal and social development outcome (Adj R^2^ = 0.51).

## Discussion

Designers of sports activities often support the assumption that young athletes' psychosocial experiences may differ because of variations in how their sport activities are designed, for example, individual or team sports. The findings of this study, in relation to the reported group differences in PNS scores between those participated in team sports and athletics versus indoor and cycling, is in line with the findings reported in the literature. For example, research has provided evidence that “youth involved in sport groups featuring greater interdependence reported greater outcomes such as enhanced developmental experiences” [61]. Furthermore, higher levels of satisfaction were reported when youth engage in activities such as team sports and games and spending time with friends [62]. Maybe the group difference in PNS reported between young athlete samples enrolled in team sports and indoor sports and cycling is in line with the assertion that working in teams is a better scenario for developmental outcome purposes than individual sports.

In the past, using various self-report measures of program quality of youth athletes, researchers have investigated the various correlates and consequences of participations in talent development programs. In this study, using a conceptual model that allows for a holistic contextual and psychological perspective on the factors associated with elite young athletes’ development, the three PNS factors, instead of the contextual factors were identified as significant predictors of developmental outcomes in the Ethiopian sports academy context. The potency of the PNS in predicting the developmental outcomes of the elite young athletes is supported in the literature on youth sports research [63].

When youth athletes involves in a positive talent development environments, it can be relevant for supporting healthy development, as well as promoting a multitude of developmental outcomes [64]. For example, in investigating the psychosocial predictors of PNS and developmental outcome, researchers reported that intrapersonal and interpersonal environmental variables accounted for a greater amount of variance in youth life satisfaction [65]. Also, another study in Brazil revealed that the satisfaction of young athletes' basic psychological needs is a strong predictor of their sports satisfaction [66]. The findings of the current study supported the argument that young athletes’ feelings of PNS would have a significant positive influence to predict developmental outcomes for young athletes in sport academy setting.

It is also evidenced that those developmental outcomes in elite young athletes are significantly positively associated with contextual and PNS factors. Several studies suggest that the ultimate goal of any youth development program should be achieving effectiveness and impacts for the individual participants and the institutions accountable for the program success [67–70]. Thus, positive predictors such as the PNS components can serve as a good indicator of the outcome of youth development programs. Hence, attention to PNS plays an important role in youth program evaluation.

## Conclusions

Based on the findings of this study, it is concluded that the talent development of elite young athletes should emphasize the individual nature of talent development processes with a significant difference across a range of sports types. Moreover, in light of the findings in this study, it is clear that young athletes who reported higher PNS would experience more positive developmental outcomes than their counterparts with lower PNS. In addition, the findings also revealed that the PNS components than the contextual factors had higher predictions of developmental outcomes, this justifies that the PNS matters more than the contextual factors. Therefore, it is relevant to have a comprehensive multidisciplinary theoretical rationale to advance understanding of the youth athletes’ talent development environment. Also, it is necessary to identify a set of interacting contextual and psychological factors associated with developmental outcomes.

### Practical implications

The novelty of this study resides in our findings that highlight the salient nature of participation in sports academies to foster PNS and promote developmental outcomes among young athletes. In youth sports education settings such as youth sports academies, it is critical to pay attention to the subtle differences in PNS among young athletes depending on the sports types. When working with young athletes in sports academies, coaches and sports psychologists must take into consideration the contextual nature and intrinsic values of PNS for the positive development of young athletes, particularly their relatedness with the environment, including instructors/coaches, fellow young athletes, or other sources of support systems. Hence, the primary target should be on making sports academies relevant to the educational, social, and emotional needs of young athletes.

### Study limitations and recommendations for future research

PNS deserves special attention for its role as a proxy for developmental outcome, as a process indicator of the quality sports academy, and ultimately as a predictor of developmental outcomes. However, the inclusion of young athletes only from two academies limits the generalizability of the findings. So, future research should employ larger and more randomized samples across different academies to help improve the generalizability and decrease bias in the design. Future research should include considerable numbers of youth coaches so that there is an increase in the overall scope of the findings. Also, this provides an opportunity for further analysis from a comparative perspective, including young athletes and coaches.

Another limitation of this study was its inclusion of a limited number of contextual factors. Future research should include more contextual information regarding socioeconomic status, parental occupation, financial resources, language spoken at home, and social support networks. This information could provide greater clarity regarding the myriad of contextual factors that contribute to the variation in PNS behaviors of young athletes and the resulted developmental outcomes.

Most studies of PNS among youth athletes, including ours looked at variables using cross-sectional data and examined between-group differences, leaving unanswered issues of temporal priority and salience, as well as within-group differences. To address these, future research should use a multivariate longitudinal study design and a multilevel modelling framework. Besides, longitudinal research designs would help assess the changes in young athletes' PNS and developmental outcomes over time.

## Data Availability

The data that support the findings of this study are available on request from the corresponding author.
